# Nuclear Entry of Activated MAPK Is Restricted in Primary Ovarian and Mammary Epithelial Cells

**DOI:** 10.1371/journal.pone.0009295

**Published:** 2010-02-18

**Authors:** Elizabeth R. Smith, Kathy Qi Cai, Jennifer L. Smedberg, Melina M. Ribeiro, Malgorzata E. Rula, Carolyn Slater, Andrew K. Godwin, Xiang-Xi Xu

**Affiliations:** 1 Department of Medicine, University of Miami Miller School of Medicine, Miami, Florida, United States of America; 2 Sylvester Comprehensive Cancer Center, University of Miami Miller School of Medicine, Miami, Florida, United States of America; 3 Ovarian Cancer and Tumor Cell Biology Programs, Department of Medical Oncology, Fox Chase Cancer Center, Philadelphia, Pennsylvania, United States of America; Bauer Research Foundation, United States of America

## Abstract

**Background:**

The MAPK/ERK1/2 serine kinases are primary mediators of the Ras mitogenic signaling pathway. Phosphorylation by MEK activates MAPK/ERK in the cytoplasm, and phospho-ERK is thought to enter the nucleus readily to modulate transcription.

**Principal Findings:**

Here, however, we observe that in primary cultures of breast and ovarian epithelial cells, phosphorylation and activation of ERK1/2 are disassociated from nuclear translocalization and transcription of downstream targets, such as c-Fos, suggesting that nuclear translocation is limited in primary cells. Accordingly, in import assays in vitro, primary cells showed a lower import activity for ERK1/2 than cancer cells, in which activated MAPK readily translocated into the nucleus and activated c-Fos expression. Primary cells express lower levels of nuclear pore complex proteins and the nuclear transport factors, importin B1 and importin 7, which may explain the limiting ERK1/2 import found in primary cells. Additionally, reduction in expression of nucleoporin 153 by siRNA targeting reduced ERK1/2 nuclear activity in cancer cells.

**Conclusion:**

ERK1/2 activation is dissociated from nuclear entry, which is a rate limiting step in primary cells and in vivo, and the restriction of nuclear entry is disrupted in transformed cells by the increased expression of nuclear pores and/or nuclear transport factors.

## Introduction

In established cell lines in culture, activation of the Ras/MAPK pathway by extracellular mitogens causes immediate, nearly complete, though transient accumulation of activated MAPK, or ERK (extracellular regulated kinase 1 and 2, or p44 and p42 ERK, respectively) in the nucleus, where it regulates transcription required for the proliferative response. One well recognized target is the transcription factor Elk-1 responsible for inducing expression of immediate early genes, including c-Fos [1], through binding to the Ets/SRE element in the c-Fos promoter [2]–[4]. The nuclear transactivating function of MAPK/ERK is absolutely required for the mitogen-stimulated growth response [1].

In vivo, however, in various organisms and model systems, the nuclear entry of activated MAPK is not unconditional but rather more stringently controlled. In the developing mouse embryo, phospho-MAPK is solely cytoplasmic in sustained, contiguous domains of MAPK signaling found in discrete areas of the embryo, yet nuclear phospho-MAPK predominates in isolated mitotic cells and in regions of the embryo that have been mechanically injured, where the cells are prompted to enter mitosis [5]. In *Drosophila*, cytoplasmic sequestration of active ERK is found in the developing eye and vein and marginal cells of the wing [6]–[9]. Phospho-MAPK remains in the cytoplasm for 2–8 hours in the R8 cells of the morphogenic furrow of the future ommotidia. A strong nuclear localization signal added to MAPK disrupts this cytoplasmic retention, and effectively upsets the differentiation pattern of the eye [7]. We have also shown that in response to retinoic acid, mouse embryonic carcinoma and stem cells undergo differentiation to embryonic primitive endoderm cells, and differentiation is accompanied by a reduction in both cell proliferation and nuclear entry of activated MAPK [10]. Cytoplasmic retention of active MAPK also occurs in senescence of human fibroblasts [11], and during cytoskeleton changes related to motility in cultured cells [12]–[14]. This evidence from multiple systems demonstrates that cytoplasmic activity of ERK/MAPK can clearly occur independently of its nuclear transcriptional function [9], [15]–[17], and nuclear restriction of phospho-ERK after cell stimulation with growth factors may be the physiologically normal circumstance in either differentiating or normal differentiated tissues.

To examine this idea, we investigated mitogen-induced translocation of activated/phospho-MAPK in primary mammary and ovarian epithelial cells and in their transformed counterparts, mammary and ovarian carcinoma cells. The newly isolated primary cells model a differentiated phenotype, whereas the transformed cells have a less differentiated, proliferative phenotype. Understanding mechanisms that distinguish normal, primary epithelial cells and/or promote the proliferation of transformed carcinoma cells may provide a site-specific target for possible prevention or therapy.

## Results

### Nuclear Entry of Activated ERK Is Limited in Primary Epithelial Cells

**Figure 1:**
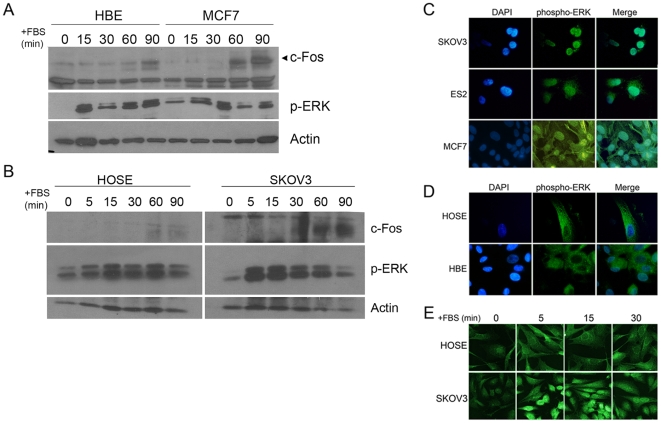
Nuclear localization of phospho-ERK is repressed in primary epithelial cells. (A) Time course of ERK activation in HBE and MCF7 cells. Primary human breast epithelial (HBE) cells and MCF7 breast carcinoma cells were serum starved for 24 h to become quiescent (time 0), then stimulated with 15% FBS for 0–90 min, and phospho-ERK (p-ERK) and c-Fos expression, a downstream marker for nuclear phospho-ERK activity, were determined in cell lysates normalized for protein. (B) Time course of ERK and cFos activation in HOSE and SKOV3 cells. Primary human ovarian surface epithelial (HOSE) cells and SKOV3 ovarian carcinoma cells were grown as described for HBE and MCF7 cells, above. Expression of c-Fos and p-ERK was determined by immunoblotting. (C-E) Primary human breast and ovarian epithelial cells at passage 1 after isolation and breast and ovarian carcinoma cells were growth arrested in serum-free medium for 24 h, then stimulated with 15% serum, and analyzed for activated ERK (phospho-ERK). Cells for immunofluorescence were fixed and probed with an anti-phosphoERK1/2 monoclonal antibody (Sigma) followed by detection using a fluorescent secondary antibody. The nuclei were counterstained with DAPI and the two images merged using Adobe Photoshop, shown in the far right column. (C) Ovarian carcinoma SKOV3 and ES2 cells and MCF7 breast carcinoma cells show robust nuclear phospho-ERK localization after serum stimulation for 15 min. (D) In cultures of primary HOSE and HBE, phospho-ERK is localized primarily to the cytoplasm after serum stimulation for 15 min. (E) Primary cultures of early passage HOSE cells and SKOV3 were stimulated with FBS for the indicated times, then processed for immunofluorescence staining and confocal imaging. The results are representative of at least 3 experiments for each cell type.

We isolated primary human ovarian surface epithelial (HOSE) cells and human breast epithelial (HBE) cells from normal tissues obtained from non-disease related surgeries. The primary cells have at least a 2-3-fold slower cell cycle than their transformed counterparts (e.g., 2-3-day versus 1-day doubling time). For all the experiments, cells were growth arrested in the G1/G0 stage of the cell cycle by incubation in serum-free medium overnight (∼24 h), then stimulated with serum for the indicated times. Serum stimulation activated MAPK equally well in primary and carcinoma cell lines, which is most apparent in the robust amount of phosphorylated ERK (p-ERK) detected in both breast (Figure 1A) and ovarian (Figure 1B) epithelial cells. In the primary HOSE cells, phospho-ERK levels equaled or surpassed those detected in SKOV3 cells in both non-synchronized, or cells grown continuously in the presence of serum, and cells stimulated with serum for 90 min (Figure S1). The downstream product of nuclear phospho-ERK activity, monitored as expression of the immediate early gene c-Fos, was weak in the primary breast and ovarian cells despite the elevated phospho-ERK levels (Figure 1A,B), and nearly undetectable in some preparations of primary cells at early passage numbers. Thus in primary epithelial cells, mitogenic stimulation does not efficiently initiate nuclear MAPK activity.

In the SKOV3 and ES2 ovarian carcinoma cells and the MCF7 breast carcinoma cell line (Figure 1C), after serum stimulation for 15 min, phospho-ERK localized predominantly to the nuclei of cells, and the intensity of immunofluorescence was fairly consistent throughout a cell population. Yet in primary HOSE and HBE cells, phospho-ERK remained principally cytoplasmic (Figure 1D). This was true at all times after serum stimulation of cells, as shown for ovarian cells (Figure 1E). Phospho-ERK could be rapidly transported into the nucleus (within 5–15 min) in both primary and transformed cells, though the magnitude and kinetics of localization differed. Fewer primary epithelial cells contained activated ERK in the nucleus compared to carcinoma cells at any time after serum addition. Nearly 90% of SKOV3 cells contained nuclear phospho-ERK compared to approximately 50% HOSE cells after 5 min serum stimulation, and the SKOV3 cells also retained nuclear phospho-ERK longer. By 30 min, approximately 65% of SKOV3 cells still retained nuclear phospho-ERK compared to only a small percentage (<10%) of HOSE cells. Similar results were found for HBE cells (not shown). Since dephosphorylation of phospho-ERK and termination of the signal presumably occurs in the nucleus [18], the primary cells appear to limit the uptake and signaling into the nucleus, and nuclear activity (e.g., c-Fos expression) correlates directly with nuclear localization of phospho-ERK in the carcinoma cells.

### MAPK Import Is Lower in Isolated Nuclei of Primary Cells

**Figure 2:**
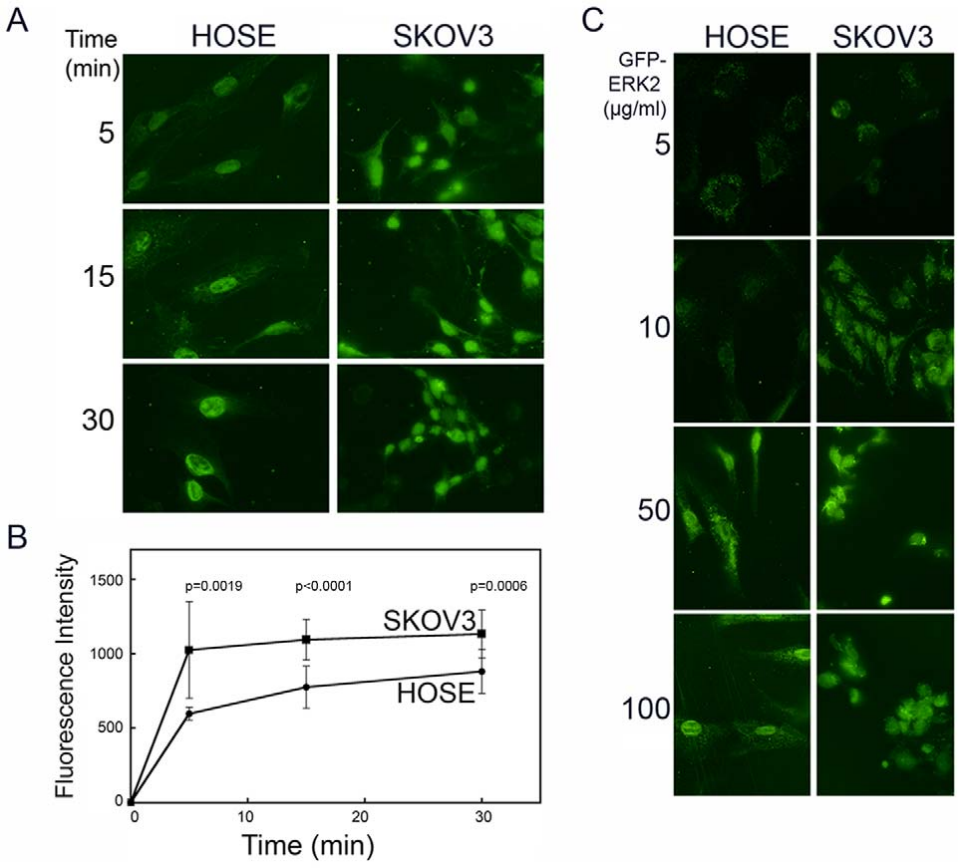
The kinetics of GFP-ERK2 nuclear import differs between primary ovarian epithelial and carcinoma cells. (A) Time course of nuclear import. Digitonin-permeabilized primary HOSE or SKOV3 carcinoma cells were incubated with GFP-ERK2 (50 µg/ml or 0.8 µM) for the indicated times, then washed in ice cold buffer and fixed in 4% paraformaldehyde. (B) Fluorescence intensity of GFP-ERK2 was measured under identical settings for the experiments shown in A. The mean relative intensity is plotted +/− s.d. of more than 50 nuclei in duplicate, and significant differences were determined by Student's t-test, with the p-values denoted above the time point. (C) The uptake of GFP-ERK2 at concentrations ranging from 5 µg/ml (0.08 µM) to 100 µg/ml (1.6 µM) was measured after 15 min incubations with digitonin-permeabilized cells, as indicated above and in “Materials and Methods.”

To understand the mechanism of nuclear restriction in primary cells, we assayed transport of His-tagged GFP-ERK2 into nuclei of primary HOSE and SKOV3 carcinoma cells using standard digitonin-permeabilized import assays [19]. The molecular mass of GFP-ERK2 (∼68 kD) is above the diffusion limit of the nuclear pore (∼40 kD), and any ERK2 tagged with GFP that enters the nucleus occurs via a non-diffusion mechanism [20]. Though GFP-ERK2 was imported into the nucleus of both SKOV3 and HOSE cells, the kinetics of accumulation differed significantly (Figure 2A,B). The intensity of nuclear GFP-ERK2 reached maximum within 5 min in SKOV3 cells, whereas it peaked after 15 min in the primary HOSE cells and did not diminish within the time course of the assay. At each time point, the difference in import between HOSE and SKOV3 was significant (p<0.005) determined by Student's t-test). Import was not energy dependent in either cell type, and in SKOV3 cells energy (in the form of an ATP/GTP regenerating system) even caused GFP-ERK2 fluorescence to fall more than 50% at longer incubations (not shown). Since both import and export of ERK occur simultaneously, this decrease or loss most likely represents GFP-ERK2 that is exported and washed out during the assay, and energy is known to be required and accelerate this export [21].

As shown in Figure 2C for HOSE cells, import does depend on the concentration of the substrate GFP-ERK2 in the assay system. At low concentrations, ERK was principally localized to the rim of the nucleus. At higher concentrations (50 and 100 µg/ml), ERK was found predominately in the nucleus. The rim staining at low concentrations indicates that ERK must interact with the nuclear pore for entry [20]. Energy did not affect the concentration-dependent import, nor were cytosolic factors absolutely required, since import occurred in assay buffer alone, without supplementation with isolated cytosol.

### Association of ERK with the Cytoskeleton Does Not Restrict Nuclear Import

Previous studies in mouse F9 embryonic carcinoma and embryonic stem cells suggested that nuclear restriction of activated ERK requires an organized, dynamic cytoskeleton [10]. We speculated the arrangement of the cytoskeleton and/or vesicles along the cytoskeleton might influence the import of ERK into the nucleus. ERK was originally identified by its ability to phosphorylate MAP2 [22], and named microtubule-associated protein-2 kinase (MAPK). Although it has been associated with the cellular cytoskeleton in a number of studies and cell systems [23], and shown to phosphorylate and regulate cytoskeletal components in vitro [13], [14], ERK is not generally or widely considered a cytoskeleton-localized protein kinase. We observed that total ERK localized in distinct filaments that extended the length of HOSE and MCF10 cells and/or outlined the perinuclear region, yet phospho-ERK was much more randomly distributed in all cells examined. Moreover, cytoskeletal-disrupting agents had negligible impact on c-Fos expression in serum-stimulated primary HBE and HOSE cells (not shown). Thus, unlike embryonic carcinoma and stem cells [10], primary epithelial cells may not require an intact cytoskeleton to limit nuclear import of activated MAPK.

### Transformation Increases Expression of Import Machinery

That MAPK is less efficiently imported into the nuclei isolated from primary ovarian epithelial cells suggested that the cells express reduced levels of nuclear import machinery compared to carcinoma cells. The major mediator of transport is the nuclear pore complex (NPC), a large pore structure through which proteins, RNAs, and other cargos move bidirectionally across the nuclear membrane. The NPC is composed of more than 50 proteins that make up the ∼125 MDa structure [24], and the central pore is lined by FG-repeat containing binding sites contributed by multiple proteins, collectively known as nucleoporins (Nup), which are believed to be responsible for mediating and specifying transport [25]. Specific cytoplasmic transport factors, known as karyopherins or importins, may also be involved in delivering proteins to the nuclear pore [26], [27].

**Figure 3:**
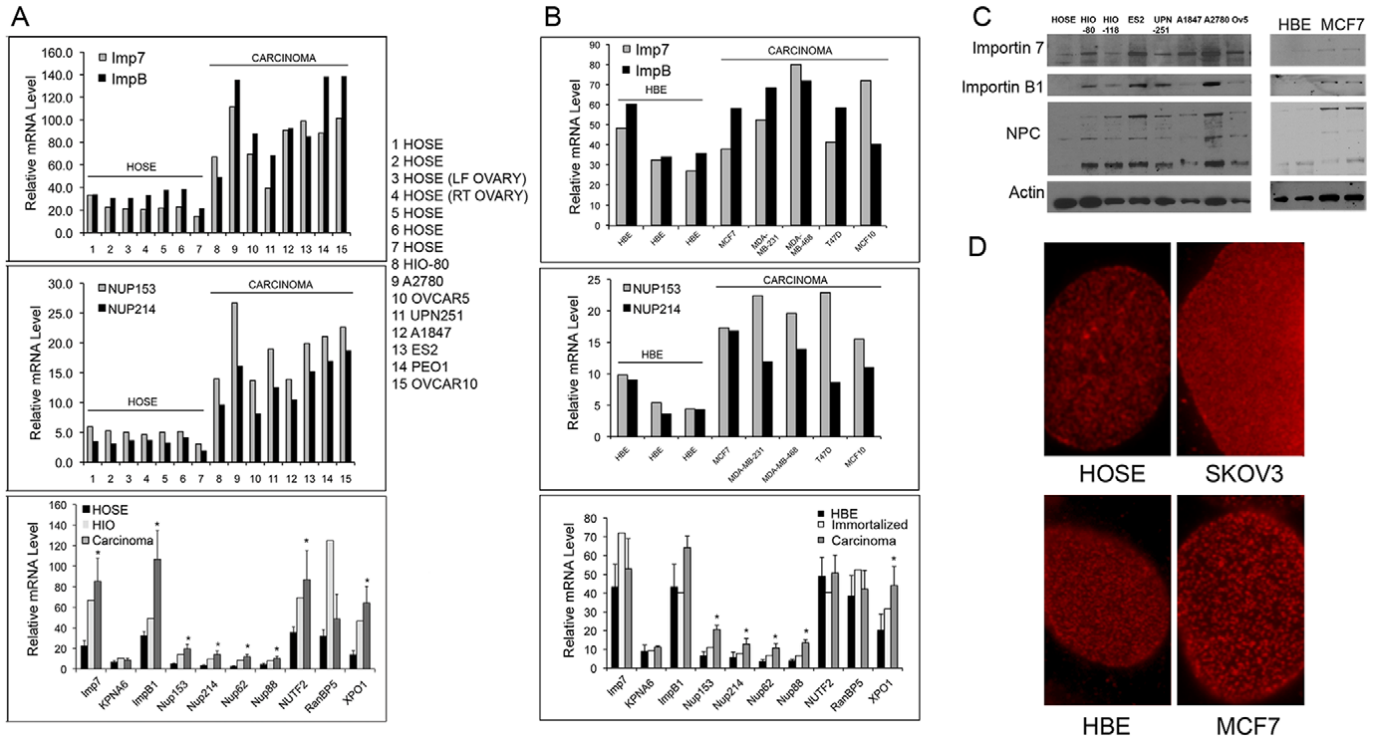
Immortalization and transformation increase transcription levels of nuclear import proteins. (A,B) The expression of a panel of nuclear import factors was examined by Nanostring nCounter methodology for ovarian (A) and breast epithelial (B) cells. (A, Top Panel) Relative mRNA levels of importin 7 (Imp7) and importin B1 (ImpB1) in normal primary HOSE, HIO, and ovarian cancer cell lines. The numbers on the x-axis correspond to the cells listed to the right of the figure. (A, Middle Panel) Expression profiles for Nup153 and Nup214 for the set of cells listed, as in the top panel. (A, Bottom panel) The expression of a panel of nuclear import factors was examined by Nanostring nCounter methodology. The data represent the mean +/− s.d. for HOSE (n = 7), HIO (n = 1), and carcinoma (n = 7) cell cultures. Differences considered statistically significant (p<0.01), calculated using Student's t-test, are indicated by an “*”. Transcripts were examined for importin 7 (Imp7), importin-alpha6 (KPNA6), importin-beta1 (ImpB1), nucleoporin 153 (Nup153), nucleoporin 214 (Nup214), nucleoporin 62 (Nup62), nucleoporin 88 (Nup88), nuclear transport factor 2 (NUTF2), Ran binding protein 5 (RNBP5), and exportin 1 (XPO1). (B) Nanostring nCounter analyses were performed for 3 HBE, the immortalized MCF10 cells, and 4 breast carcinoma cell lines (MCF7, MDA-MB-231, MDA-MB-468, T47D), as in Figure 3A. (C) Immunoblot analyses were performed for Importin 7, Importin B, and NPC in primary HOSE and HBE cells, human immortalized ovarian epithelial (HIO) cells, MCF7 breast carcinoma cells, and a panel of ovarian carcinoma cells (ES2, UPN251, A1847, A2780, and Ovcar5). NPC proteins were detected with a pan-anti-NPC mouse monoclonal antibody (from Sigma). (D) Primary HOSE and HBE cells and SKOV3 and MCF7 carcinoma cells were fixed and stained for nuclear pores using a pan-anti-NPC mouse monoclonal antibody and an Alexa555-conjugated goat anti-mouse secondary antibody. All images were treated identically using Adobe Photoshop.

Nano-String gene expression profiling [28] confirmed that nucleoporins and import transport factors were elevated in ovarian and breast cancer cells at the transcript level (Figure 3). Seven different HOSE cell preparations (for Nano-String profiling) showed similar mRNA levels, whereas the transformed cells consistently had elevated message levels of NPC proteins (Figure 3A, middle and lower panels). ERK has been shown to interact with nucleoporin-214 (Nup214) and nucleoporin-153 (Nup153) through FG-repeat sequences [20], [29]. Moreover, the ovarian cancer cells typically had higher transcript levels of importin 7 (Imp7) and importin B1 (ImpB1, also known as KPNB1), members of the karyopherin/importin-beta family of nuclear transport factors that have been shown in *Drosophila* to modulate MAPK/ERK nuclear localization [8], [9], [30], [31]. The transcript levels of importin-alpha (KPNA6) and RanBP5 cytosolic transport factors changed minimally. The distinction was apparent even between human immortalized ovarian (HIO) epithelial cells and the primary HOSE cells (Figure 3A, lower panel). Originally generated by transfecting HOSE cells with the SV40 large T antigen, the HIO cells are non-tumorigenic but can be cultured up to 30 passages [32], [33]. We also examined mRNA levels in three HBE, the immortalized MCF10 cell line, and four breast carcinoma cell lines, and observed that gene expression for nucleoporins was significantly elevated (p<0.01) in all breast cancer cells relative to the primary cells (Figure 3B), which followed a trend similar to ovarian cancer and ovarian primary cells. Importin levels were reduced significantly in two of the three primary HBE lines (Figure 3B, top panel), though the average mRNA expression level of the three HBE lines did not appear significantly different from that found in the breast carcinoma cells. Northern blots for importin B1 and Nup153 confirmed that ovarian carcinoma and HIO cells contained increased message levels for these genes compared to normal HOSE cells (not shown). Moreover, immunoblotting confirmed that nucleoporins and import transport factors were elevated at the protein level in the carcinoma cells (Figure 3C). The increased expression of nucleoporins Nup153 and Nup214 and importin-beta factors strongly predicts elevated MAPK nuclear import in tumor tissues.

Nevertheless, assembly of the nuclear pore does not require expression of all nucleoporins, or an uncompromising stoichiometry of individual nucleoporins [34], [35]. To verify that the expression level of nucleoporins correlated with the number of intact pores, we evaluated the density of pores per nuclei compared to carcinoma cells by immunofluorescence microscopy. Nuclear pores stained with NPC appear as distinct punctate spots over the nuclear surface and staining is more dense and/or intense in the carcinoma cells (Figure 3D). For example, the density of NPC in SKOV3 cells is consistently greater than HOSE cell, although the actual intensity of the individual pores appears to be more prominent than the density in MCF7 cells compared to normal breast epithelial cells. Counting of the individual pores present in confocal images taken at the greatest cross-sectional diameter of the nuclei suggested that the carcinoma cells contained approximately 20–30% more pores than the normal primary cells, yet the difference in the number of pores is less than would be predicted based on the difference in transcript and expression level. Thus, the stoichiometry of nucleoporins in the nuclear pore, as well as the pore number, may contribute to the differences observed in message and protein content in cancer cells.

**Figure 4:**
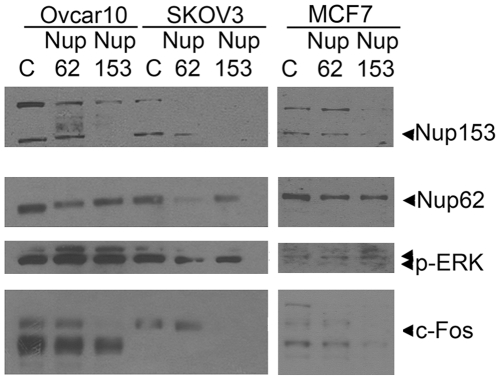
Suppressed expression of nucleoporins decreases c-Fos expression in carcinoma cells. OVCAR-10 and SKOV3 ovarian and MCF7 breast cancer cells were transiently transfected with siRNA constructs to Nup153 and Nup62, as well as a control siRNA (from Santa Cruz). After 48 h, cells were serum-starved overnight, then stimulated with 20% FBS for 90 min, and lysed for immunoblot analysis. NPC proteins were detected with a mouse pan-anti-NPC monoclonal antibody.

We used siRNAs targeted against Nup153 and as a control Nup62, another FG repeat-containing central pore protein that has not been reported to interact with phospho-ERK, to analyze the downstream effect on nuclear ERK activity. Consistently, down-regulation of Nup153 decreased c-Fos expression in transfected cells without lowering phospho-ERK levels (Figure 4), showing that uncoupling of MAPK activation and c-Fos expression occurs at the level of nuclear import machinery. As predicted, changes in Nup62 levels did not affect c-Fos expression.

### Expression of NPC Is Increased in Carcinomas

**Figure 5:**
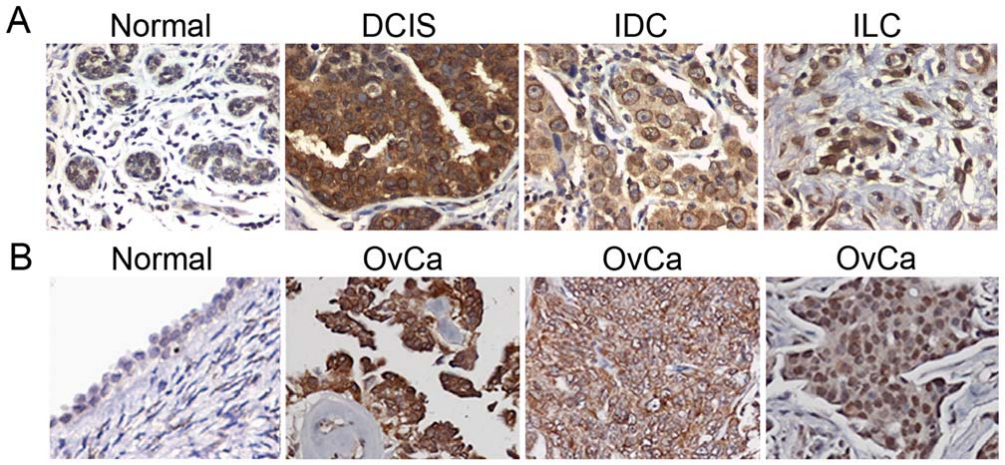
NPC expression is upregulated in breast and ovarian carcinomas. (A) Immunohistochemical analysis of NPC expression was undertaken on a tumor tissue microarray (TMA) containing normal breast epithelial tissue and tumor specimens, including DCIS (ductal carcinoma in situ), IDC (infiltrating ductal carcinoma), and ILC (infiltrating lobular carcinoma). A mouse pan-anti-NPC monoclonal antibody was used for immunostaining. (B) Immunohistochemical staining of NPC on ovarian TMA containing three ovarian serous carcinomas (OvCa) compared to a normal ovarian surface epithelium (left panel). Images were taken at 400× magnification.

Since we observed that the level of NPC is elevated in transformed cells in culture, we next examined whether NPC levels were increased in tumor tissues. Staining of ovarian and breast tumor specimens on several tissue microarrays (TMA) indicated expression of NPC was elevated in most transformed tissues (Figure 5) (see Tables S1, S2, and S3 for information and pathology of ovarian and breast cancers on the TMAs). The majority of breast tumor tissues stained positive for NPC (Figure 5A). In the informative tissue cores, the intensity of NPC staining was typically pervasive (70% of the epithelial component was positive) and strong (intensity was 2–3) in the DCIS (ductal carcinoma in situ) specimens. Normal breast epithelial tissue had weak (0–1) staining in an average 50% (range 40–70%) of the epithelial cells. The more malignant type carcinomas, IDC (infiltrating ductal carcinoma) and ILC (infiltrating lobular carcinoma), had more variable NPC staining (Figure 5A), though in general, the intensity was low to medium (Tables S1, S3). Thus, over-expression of NPC may correlate more closely with DCIS than the other tumor types; however, the sample size is small and would need to be increased substantially to reach a conclusion.

In the ovarian cancer microarrays examined, NPC staining reflected the heterogeneous nature of ovarian carcinoma, with a range of epithelial components scoring positive (20–90%), and no clear correlation could be found between tumor type, stage, or grade. However, out of the panel of informative specimens (those that had an epithelial component that could be scored for staining), over 60% of the ovarian cancer samples stained 80% or greater of the tumor epithelium highly positive for NPC (intensity 2–3) (Tables S2, S3). Figure 5B shows examples of a normal ovarian surface epithelium (left panel, denoted by the arrow) and three cases of ovarian serous adenocarcinoma, the most predominant ovarian tumor type. Though the normal ovarian epithelia stained positively for NPC, the intensity of staining was characteristically much weaker (intensity 1–2).

Thus, we observed that a large proportion of breast and ovarian tumors express an increased level of NPC. It is quite possible that the tumors in which NPC is not over-expressed, importin transport factors, such as importin B and importin 7, may well be, though available antibodies are not yet suitable for immunohistochemical staining of tissues.

## Discussion

Although the Ras/MAPK pathway has been investigated extensively in mammalian cells, many key observations have been made using continuously cultured, transformed cell lines. Mitogen stimulation of these cells results in a significant portion of phospho-MAPK accumulating transiently in the nucleus. Here we report that primary epithelial cells restrict the amount of phospho-ERK localized in the nucleus after pathway stimulation, although the cells obviously have an intact Ras/MAPK signaling pathway and the means to activate ERK. In fact, the actual time course of activation between primary and carcinoma cell lines is remarkably similar, though primary cells maintain higher levels of activated ERK longer following mitogenic stimulation, which would be expected since termination of the signal by MAPK-specific phosphatase MKP1 and MKP2 occurs in the nucleus [18]. The effect of this restriction is to limit the proliferation of the cells or tissues and maintain the differentiated phenotype.

Primary epithelial cells, such as HOSE, require EGF and other serum factors for maintenance, growth, and clonal propagation in culture, as shown in early studies designed to configure growth conditions for isolated cells [36]–[38], and these cells express the EGF receptor both in vivo and in culture [39]. The ovarian surface epithelial cells are believed to proliferate slowly in the intact organ [40], yet these cells must proliferate to cover the site where ovulation has occurred. Follicle rupture simulates a physical injury, which forces the cells in the area to enter mitosis, and several rounds of division occur to repair the wound [41]. This is very similar to the findings made in the mouse embryo, where wounding or scraping stimulates an accumulation of phospho-MAPK in the nucleus [5], and suggest that an event(s) or factor(s) separate from or in addition to growth factor stimulation is required to force nuclear translocation of phospho-MAPK in the primary cells. These factors could include cytoskeletal rearrangements and expression, calcium fluxes, cell interactions with stroma, matrix, or other cells, shape changes, altered expression of import proteins, or a specific phosphorylation signal on ERK [42]. An increase in the local concentration of EGF via release from platelets recruited to the wound site after ovulation may be involved [38], [43], as well as the local microenvironment [40].

It is likely that the mechanism responsible for MAPK nuclear restriction may differ between cells and tissue types or developmental stages. Preliminary data in embryonic carcinoma cells suggests that importin B down-regulation may suppress c-Fos expression following serum stimulation and MAPK activation (unpublished observations). Furthermore, both importin 7 (DIM7/MSK) and importin B (Ketel) were originally found to affect MAPK nuclear import in *Drosophila* [30]. In some specific phases of eye development, importin 7/MSK seems to restrict, rather than promote, nuclear import of MAPK by its being sequestered apically and thereby holding phospho-MAPK in the cytoplasm, promoting differentiation over proliferation [8], [9]. Another report, however, suggests that activation of MAPK must be spatially coupled by integrin-mediated phosphorylation of importin 7/MSK, and this activated complex permits MAPK to be imported efficiently [31]. In this latter study, changes in MAPK phosphorylation were not examined. Thus, how these multiple signals are initiated and specified to regulate MAPK are obvious important questions to investigate.

The results from this study show that global changes in expression of nuclear transport components occur with immortalization and transformation of breast and ovarian epithelial cells, as indicated by the increase in NPC, as well as importin B and importin 7, which would modulate entrance of a cargo (such as MAPK) to the pore itself. More importantly, enhanced nuclear transport or permeability may be permissive or alternatively required for transformation. Specific nucleoporin levels apparently regulate cell cycle progression and phase-specific gene expression [44], and expression of individual pore and transport factor proteins can differ between tissues and developmental stages [24], [35], [45], and in several disease states, where over-expression of importin-alpha and –beta have been reported in colon, breast, and lung cancers [24], [35]. Our findings of increased NPC staining in ovarian and mammary tumors substantiate this, and expression profiling confirmed an early observation that mRNA for Nup88 was over-expressed in a panel of ovarian carcinoma cell lines [46]. A lowered level of importin 7 and in particular importin B would be predicted to reduce the efficiency of MAPK nuclear import in the primary cells, as it has been found in *Drosophila* and other cell types [8], [47], [48]. Moreover, the number of functional pores is known to vary depending upon the growth state of the cell, increasing in proliferating versus quiescent BALB/c 3T3 cells [45].

The interaction of MAPK with the nuclear pore is obviously specific, since down-regulation of Nup153, but not Nup62, affected c-Fos expression. Nup62 exists in a subcomplex with three other nucleoporins (Nup58, Nup54, and Nup62) that is located at both the cytoplasmic and nuclear periphery of the central core of the pore, which is embedded in the nuclear membrane and sandwiched between a cytoplasmic and nuclear ring. The cytoplasmic ring has eight fibrils that extend into the cytoplasm; the nuclear ring carries eight fibrils that form a basket-like structure that extends into the nucleoplasm. Nup153 faces the midsection of the nuclear basket [24], [25], [35], [49]. It is known that general nuclear transport remains efficient even when changes occur in the pore composition, and changes in Nup153 appear to modulate modestly NLS-mediated import [50]. As we observed, however, suppression of Nup153 significantly alters specific MAPK signaling dependent upon its nuclear import.

In summary, we conclude that ERK1/2 nuclear entry is a rate limiting step in primary cells and in vivo, and the restriction of nuclear entry is abolished in transformed cells by increased expression of nuclear pores and/or nuclear transport factors.

## Materials and Methods

### Ethics Statement

Primary tumor and non-tumor tissue samples were obtained from patients who underwent surgical resection at Fox Chase Cancer Center. Tumor microarrays (TMAs) were constructed from the tumor collection held by the Department of Pathology at Fox Chase Cancer Center. Tumor grades and histological subtypes were obtained from the pathology reports without reference to the patients' personal information. The use of these human tissues was examined and approved by the Fox Chase Cancer Center institutional Human Investigation Committee, and safety and ethical guidelines were followed. Written informed consent was obtained from each patient, and extensive precautions were taken to preserve the privacy of the participants donating tissue. Each patient sample was assigned a unique study code, the only source of identification visible on the biospecimens and any accompanying paperwork. Our work was performed blinded, without reference to patient sample number.

### Antibodies

Antibodies were obtained from multiple sources and used at the specified dilutions for immunofluorescence microscopy: activated diphosphorylated Erk2/1 mouse monoclonal (Clone MAPK-YT, Sigma) 1∶200; phospho-p44/42 MAP kinase rabbit polyclonal and MAP kinase rabbit polyclonal (Cell Signaling Technology) 1∶250; Erk1 mouse monoclonal (BD Transduction Lab) 1∶50. For immunoblots, antibodies were used at the following dilutions: NPC proteins mouse monoclonal (Sigma) 1∶5000; Importin B1 mouse monoclonal (Affinity Bioreagents) 1∶5000; Importin 7 goat polyclonal (Imgenex) 1∶1000; c-Fos rabbit polyclonal (Santa Cruz) 1∶1000; activated diphosphorylated Erk2/1 (Sigma) 1∶5000; beta-actin mouse monoclonal (Sigma) 1∶5000. Alexa-488-conjugated, Alexa-555, and Rhodamine Red-conjugated secondary antibodies came from Molecular Probes.

### Preparation and Culture of Primary Ovarian Surface Epithelial Cells

Isolation of human ovarian surface epithelial cells is based on the relatively loose attachment of surface ovarian epithelial cells to underlying structures [3], [37]. Cells were prepared from intact human ovaries of non-diseased patients, obtained with informed consent, following oophorectomies at Fox Chase Cancer Center. The institutional Human Investigation Review Board of Fox Chase Cancer Center fully approved the use of the samples and making of cell lines. The cells were cultured in flasks coated with 0.1% swine skin gelatin in Medium 199/MCCB 105 (1∶1 ratio) containing 15% FBS, penicillin and streptomycin, 2 mM L-glutamine, and 0.25 U/ml insulin. In some later experiments, medium also contained bovine pituitary extract. Expansion of HOSE cells typically begins 7–14 days after isolation, and the cells can be subcultured 10–15 times before undergoing replicative senescence [38]. Human immortalized ovarian surface epithelial cell lines were derived and characterized as described previously [32], [33]. Ovarian tumor cell lines were previously established [41] or obtained from ATCC (Rockville, MD). The tumor cells were cultured in RPMI containing 10% FBS and 1X antibiotic/antimycotic solution (Gibco). Carcinoma cell lines were transfected with 20 nM siRNA constructs (Santa Cruz) for 48 h, using Lipofectamine 2000 according to the manufacturer's protocol (Invitrogen).

### Isolation and Culture of Primary Mammary Epithelial Cells

Epithelial cells were obtained as outgrowths of normal breast tissue acquired from breast reduction surgery and digested to epithelial organoids, as described [51], [52]. Cell cultures were maintained at a high density on tissue culture flasks coated with 0.1% swine skin gelatin in DMEM/F12 supplemented with 5% horse serum, 20 ng/ml EGF, 100 ng/ml cholera toxin, 10 mg/l insulin, 0.5 mg/l hydrocortisone, 0.04 mM calcium chloride, and 1X antibiotic/antimycotic, and split 1∶1 or 1∶2 when confluent. Active growth of these cells generally begins 3–4 days after seeding and maintained for approximately 10 passages, with subculture every 7–14 days [51]. In some cases, breast epithelial cells were frozen in 90% FBS/10% DMSO, and cultures were re-established from frozen stocks.

### Recombinant Proteins and Nuclear Import Assays

The cDNA encoding rat His-tagged GFP-ERK2 was a generous gift of Dr. Melanie Cobb (University of Texas Southwestern Medical Center) and was prepared as described [53]. In this construct the rat ERK2 cDNA was subcloned into pRSETB-His_6_-GFP using KpnI and HindIII restriction sites. Preparation and purification of GFP-ERK2 followed the published procedures [53]. Import assays were performed using HOSE or SKOV3 cells plated on gelatinized glass coverslips in 6-well dishes [19], [54]. Cells were plated on coverslips at least 2 days before the assay and incubated in serum-free culture medium at least 24 h before nuclear import of GFP-ERK2 was assayed.

### NanoString nCounter Expression Analysis

The mRNA content of cell lysates was analyzed by NanoString methodology according to published procedures [28], and was performed by the Oncogenomic Core Facility at the UM-Sylvester Comprehesive Cancer Center. Briefly, 100 ng of total RNA per replicate or lysate of 10,000 cells in Qiagen RLT lysis buffer were hybridized to the target specific codeset ON at 65°C. The codeset contained probes against a panel of 13 genes encoding proteins involved in nuclear import. The hybridized reactions were loaded onto the NanoString Prep station, which removes excess reporter, binds the reporter to the cartridge surface, and stretches the probes for scanning. Subsequently, the cartridges were loaded onto the NanoString Digital Analyzer and scanned. Nanominer software was used to perform normalization compared to Actin and basic statistical analysis on the data. The normalized results are expressed as the relative mRNA level, and values for normal and carcinoma cells were averaged and shown as mean ± s.d. Statistical significance was calculated using Student's t-test and was set as p<0.01.

### Immunostaining

Immunological staining was performed using the mouse DAKO Envision TM+ System and the peroxidase (DAB) kit (DAKO Corporation, Carpineria, CA) as described [55]. Tumor microarrays were constructed from the tumor collection held by the Department of Pathology at Fox Chase Cancer Center. Tumor grades and histological subtypes were obtained from the pathology reports without reference to the patients' personal information.

### Immunofluorescence Microscopy

Cells were grown on glass coverslips in 6-well dishes in the appropriate culture medium. After treatment, cells were washed once with PBS at room temperature and fixed in 4% paraformaldehyde for 10 min, lysed with 0.1% Triton X-100 in PBS for 5 min at room temperature, and processed for immunofluorescence [10]. Slides were viewed on a Nikon Eclipse TE300 microscope using a 100× oil-immersion objective, coupled to a Roper Scientific CoolSnap ES camera, and analyzed with MetaVue (Universal Imaging/Molecular Devices) or NIS-Elements Basic Research (Nikon) and Adobe Photoshop software. Alternatively some fixed and stained cells were viewed on a Nikon E800 upright microscope equipped with a BioRad 2000 confocal scanhead using a 60× oil-immersion objective, and images were deconvoluted using the Laser Sharp2000 (Zeiss) and MetaVue software and analyzed using Adobe Photoshop. To quantitate NPC, confocal images were taken on a Zeiss LSM ZeissLSM510 Confocal laser-scanning microscope using 63× oil objective (N/A 1.4), and the section with the widest cross-section of the nucleus selected for further analysis. All images were zoomed using Adobe Photoshop and treated identically.

### Cytoskeleton Isolation

Cytoskeleton was prepared in microtubule stabilizing buffer (MSB) with the addition of protease inhibitors (Complete Mini, Roche). Cells were plated on glass coverslips in 6-well dishes. Following treatment, cells were washed two times in MSB (0.1 M Pipes, pH 6.9, adjusted with KOH, 1 mM EDTA, 4% glycerol, 1 mM sodium orthovanadate). Soluble proteins were extracted for 5 min in MSB containing 0.2% Triton X-100. The dishes were then washed twice with MSB, and cytoskeletons were fixed at room temperature in 4% paraformaldehyde in PBS.

## Supporting Information

Figure S1 Primary HOSE and SKOV3 ovarian carcinoma cells were growth arrested overnight, then stimulated with serum for 0 or 90 min, or continuously grown in the presence of serum (NS, non-synchronized). Lysates were analyzed for c-Fos and phospho-ERK (p-ERK) expression.
(4.15 MB TIF)

Table S1 NPC immunostaining of breast tumor tissue microarray. The breast tumor tissue microarray (06-01) was stained for NPC by immunohistochemistry. The data shown in Table S3 was tabulated according to the percent of the total number of samples for each tumor type staining low (+), medium (++), or high (+++) for NPC. Seven of nine normal breast tissue samples contained sufficient epithelial component to score.
(0.03 MB DOC)

Table S2 NPC immunostaining of ovarian tumor tissue microarrays. The ovarian tumor tissue microarrays were stained for NPC by immunohistochemistry. The data shown in Table S3 was tabulated according to the percent of the total number of samples for each tumor type staining low (+), medium (++), or high (+++) for NPC. Only two of the seven normal tissue samples contained sufficient epithelial component to score.
(0.05 MB DOC)

Table S3 Immunostaining of ovarian and breast cancer tissue microarrays. Breast and ovarian tumor tissue microarrays were stained for NPC by immunohistochemistry. Scoring of NPC staining represents the % of epithelial cells that were positive for NPC, whereas intensity is scored from undetectable (0), low (+), medium (++), to high (+++). Some cases contained an insufficient epithelial component and are left unscored (---).
(0.16 MB DOC)
