# Supplementary figures and images for: Nuclear Entry of Activated MAPK Is Restricted in Primary Ovarian and Mammary Epithelial Cells
Source: PLoS One. 2010 Feb 18;5(2):e9295. doi: 10.1371/journal.pone.0009295 (PMC2823791; doi:10.1371/journal.pone.0009295)

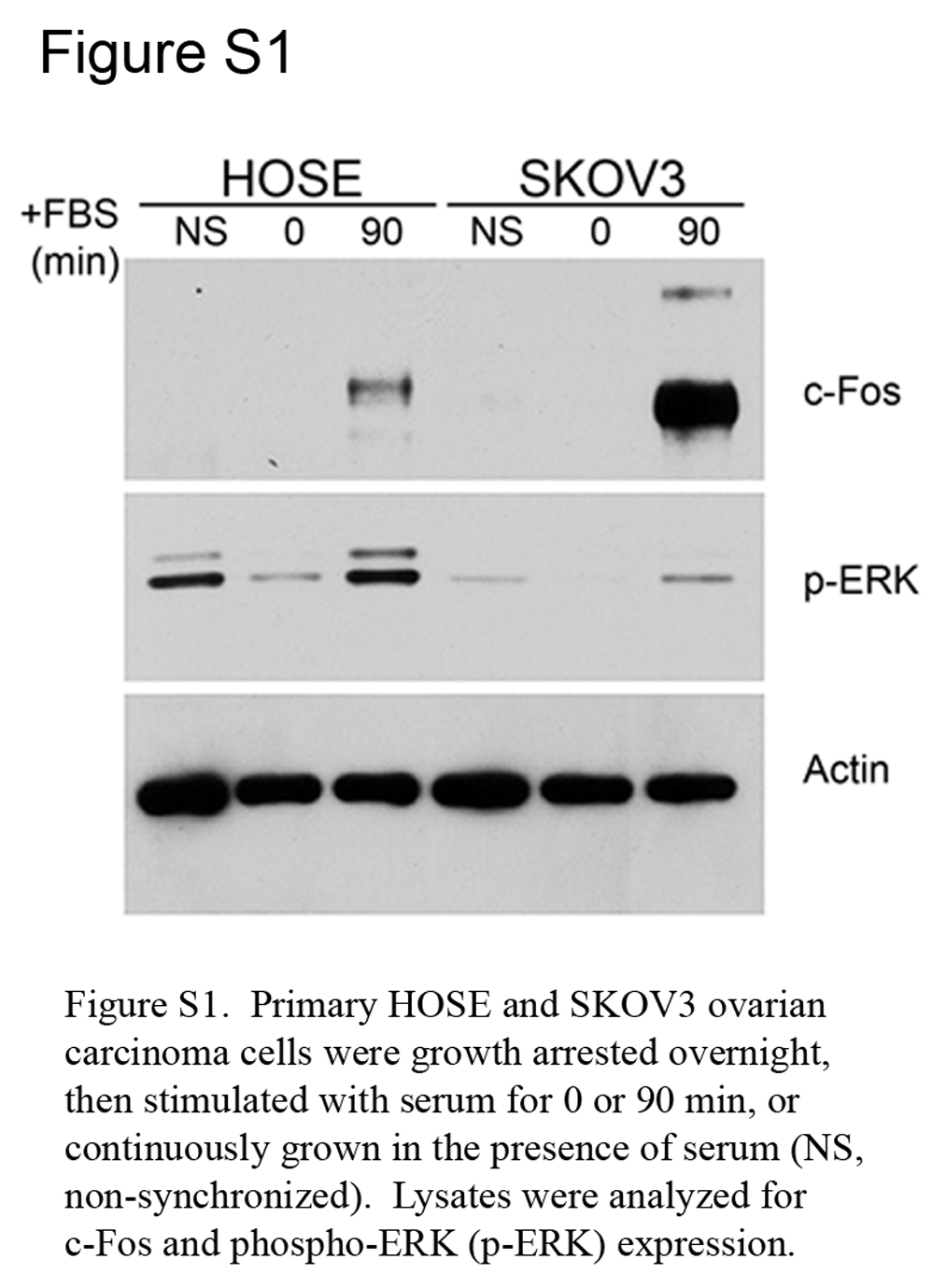

Supplement: Figure S1 — Primary HOSE and SKOV3 ovarian carcinoma cells were growth arrested overnight, then stimulated with serum for 0 or 90 min, or continuously grown in the presence of serum (NS, non-synchronized). Lysates were analyzed for c-Fos and phospho-ERK (p-ERK) expression. (4.15 MB TIF) [file pone.0009295.s001.tif]
