# Supplementary material for: Nuclear Entry of Activated MAPK Is Restricted in Primary Ovarian and Mammary Epithelial Cells
Source: PLoS One. 2010 Feb 18;5(2):e9295. doi: 10.1371/journal.pone.0009295 (PMC2823791; doi:10.1371/journal.pone.0009295)
Supplement: Table S1 — NPC immunostaining of breast tumor tissue microarray. The breast tumor tissue microarray (06-01) was stained for NPC by immunohistochemistry. The data shown in Table S3 was tabulated according to the percent of the total number of samples for each tumor type staining low (+), medium (++), or high (+++) for NPC. Seven of nine normal breast tissue samples contained sufficient epithelial component to score. (0.03 MB DOC) [file pone.0009295.s002.doc]

**Table S1. NPC Immunostaining of Breast Tumor Tissue Microarray**

The breast tumor tissue microarray (06-01) was stained for NPC by immunohistochemistry. The data shown in Supplemental Table S3 was tabulated according to the percent of the total number of samples for each tumor type staining low (+), medium (++), or high (+++) for NPC. Seven of nine normal breast tissue samples contained sufficient epithelial component to score.

**NPC Staining Intensity**

Number (% Total)

| **Tissue type** | **Total No. Samples** | **Undetectable** | **Low** | **Medium** | **High** |
| --- | --- | --- | --- | --- | --- |
|  |  |  |  |  |  |
| **Normal** | 7 | 1 (14%) | 6 (86%) | 0 | 0 |
|  |  |  |  |  |  |
| **DCIS** | 8 | 2 (25%) | 3 (37.5%) | 1 (12.5%) | 3 (37.5%) |
|  |  |  |  |  |  |
| **IDC** | 7 | 1 (14%) | 3 (43%) | 2 (29%) | 1 (14%) |
|  |  |  |  |  |  |
| **ILC** | 2 | 1 (25%) | 1 (12.5%) | 3 (37.5%) | 2 (25%) |
