# Supplementary material for: Nuclear Entry of Activated MAPK Is Restricted in Primary Ovarian and Mammary Epithelial Cells
Source: PLoS One. 2010 Feb 18;5(2):e9295. doi: 10.1371/journal.pone.0009295 (PMC2823791; doi:10.1371/journal.pone.0009295)
Supplement: Table S2 — NPC immunostaining of ovarian tumor tissue microarrays. The ovarian tumor tissue microarrays were stained for NPC by immunohistochemistry. The data shown in Table S3 was tabulated according to the percent of the total number of samples for each tumor type staining low (+), medium (++), or high (+++) for NPC. Only two of the seven normal tissue samples contained sufficient epithelial component to score. (0.05 MB DOC) [file pone.0009295.s003.doc]

**Table S2. NPC Immunostaining of Ovarian Tumor Tissue Microarrays**

The ovarian tumor tissue microarrays were stained for NPC by immunohistochemistry. The data shown in Supplemental Table S3 was tabulated according to the percent of the total number of samples for each tumor type staining low (+), medium (++), or high (+++) for NPC. Only two of the seven normal tissue samples contained sufficient epithelial component to score.

**NPC staining intensity**

Number (% total)

| **Tissue type** | **Total No. Samples** | **Undetectable** | **Low** | **Medium** | **High** |
| --- | --- | --- | --- | --- | --- |
|  |  |  |  |  |  |
| **Ser Surf Pap Ca** | 3 | 0 | 0 | 0 | 3 (100%) |
|  |  |  |  |  |  |
| **Pap Ser Adeno Ca** | 32 | 0 | 2 (6.25%) | 8 (25%) | 22 (68.75%) |
|  |  |  |  |  |  |
| **Pap Ser Cystadenoma** | 5 | 0 | 1 (20%) | 0 | 4 (80%) |
|  |  |  |  |  |  |
| **Ser Adeno Ca** | 3 | 0 | 1 (33.3%) | 1 (33.3%) | 1 (33.3%) |
|  |  |  |  |  |  |
| **Pap Ser Ca** | 9 | 0 | 1 (11%) | 3 (33%) | 5 (56%) |
|  |  |  |  |  |  |
| **Adeno Ca** | 4 | 0 | 0 | 3 (75%) | 1 (25%) |
|  |  |  |  |  |  |
| **Pap Adeno Ca** | 1 | 0 | 0 | 1 (100%) | 0 |
|  |  |  |  |  |  |
| **Clear Cell Adeno Ca** | 3 | 0 | 0 | 1 (33%) | 2 (67%) |
|  |  |  |  |  |  |
| **Met Ovarian Adeno Ca** | 3 | 0 | 0 | 0 | 3 (100%) |
|  |  |  |  |  |  |
| **Mucinous** | 3 | 0 | 1 (33%) | 2 (67%) | 0 |
|  |  |  |  |  |  |
| **Mixed Mesodermal** | 1 | 0 | 0 | 1 (100%) | 0 |
|  |  |  |  |  |  |
| **Normal** | 2 | 0 | 1 (50%) | 1 (50%) | 0 |
