# Supplementary material for: Nuclear Entry of Activated MAPK Is Restricted in Primary Ovarian and Mammary Epithelial Cells
Source: PLoS One. 2010 Feb 18;5(2):e9295. doi: 10.1371/journal.pone.0009295 (PMC2823791; doi:10.1371/journal.pone.0009295)
Supplement: Table S3 — Immunostaining of ovarian and breast cancer tissue microarrays. Breast and ovarian tumor tissue microarrays were stained for NPC by immunohistochemistry. Scoring of NPC staining represents the % of epithelial cells that were positive for NPC, whereas intensity is scored from undetectable (0), low (+), medium (++), to high (+++). Some cases contained an insufficient epithelial component and are left unscored (---). (0.16 MB DOC) [file pone.0009295.s004.doc]

**Table S3. Immunostaining of ovarian and breast cancer tissue microarrays.**

Breast and ovarian tumor tissue microarrays were stained for NPC by immunohistochemistry. Scoring of NPC staining represents the % of epithelial cells that were positive for NPC, whereas intensity is scored from undetectable (0), low (+), medium (++), to high (+++). Some cases contained an insufficient epithelial component and are left unscored (---).

| **Breast TMA 06-01** | | **NPC** | |
| --- | --- | --- | --- |
| **GRID** | **DIAGNOSIS** | **%** | **INTENSITY** |
| B3-B4 | DCIS | 70% | +++ |
| B5-B6 | Normal | 50-60% | + |
| B7-B8 | DCIS | 70% | +++ |
| B9-B10 | DCIS | 70% | +++ |
| C3-C4 | DCIS | 0 | 0 |
| C5-C6 | DCIS | 0 | 0 |
| C7-C8 | Normal | 70% | + |
| C9-C10 | DCIS | 70% | +~++ |
| D3-D4 | DCIS | 70~80% | + |
| D5-D6 | DCIS | 70~80% | + |
| D7-D8 | Normal | 70-80% | 0~+ |
| D9-D10 | IDC | 80% | +++ |
| E3-E4 | IDC | 50% | + |
| E5-E6 | Normal | --- | --- |
| E7-E8 | IDC | 60~70% | + |
| E9-E10 | IDC | 60% | ++ |
| F3-F4 | IDC | 80% | + |
| F5-F6 | Normal | 0 | 0 |
| F7-F8 | Normal | --- | --- |
| F9-F10 | IDC | 70% | ++ |
| G3-G4 | IDC | 0 | 0 |
| G5-G6 | Normal | 50% | 0~+ |
| G7-G8 | ILC | 60% | ++~+++ |
| G9-G10 | ILC | 0 | 0 |
| H3-H4 | ILC | 0 | 0 |
| H5-H6 | ILC | 50~60% | 0~+ |
| H7-H8 | Normal | 40~50% | 0~+ |
| H9-H10 | ILC | 60~70% | ++ |
| I3-I4 | ILC | 50~60% | ++ |
| I5-I6 | Normal | 60% | + |
| I7-I8 | ILC | 80% | +++ |
| I9-I10 | ILC | 60% | +~++ |

DCIS, ductal carcinoma in situ

IDC, infiltrating ductal carcinoma

ILC, infiltrating lobular carcinoma

| **Ovarian TMA-03-02** | | | | **NPC** | |
| --- | --- | --- | --- | --- | --- |
| **GRID** | **DIAGNOSIS** | **GRADE** | **STAGE** | **%** | **INTENSITY** |
| B3-B4 | Ser Surface Pap Ca | 3 | 3C | 90% | +++ |
| B5-B6 | Pap Ser Adeno Ca | 3 | 4 | --- | --- |
| B7 |  |  |  |  |  |
| B8-B9 | Pap Ser Cystadenocarcinoma | 2 | 3B | 40% | + |
| B10-B11 | Pap Ser Adeno Ca | 2 | 3C | 50% | + |
| C3-C4 | Pap Ser Adeno Ca | 2 | 3C | 70% | +++ |
| C5 |  |  |  |  |  |
| C6-C7 | Pap Ser Adeno Ca | 3 | 3C | 70% | ++~+++ |
| C8-C9 | Pap Ser Adeno Ca | 3 | 3C | 70% | +++ |
| C10-C11 | Pap Ser Adeno Ca | 3 | 3C | --- | --- |
| D3-D4 | Pap Ser Cystadenocarcinoma | 3 | 3C | 90% | +++ |
| D5-D6 | Pap Ser Adeno Ca | 3 | 4 | 90% | +++ |
| D7-D8 | Pap Ser Adeno Ca | 3 | 2B | 90% | +++ |
| D9 |  |  |  |  |  |
| D10-D11 | Pap Ser Adeno Ca | 3 | 3C | 80% | ++ |
| E3-E4 | Pap Ser Cystadenocarcinoma | 3 | 3C | 90% | +++ |
| E5-E6 | Pap Ser Adeno Ca | 3 | 3C | 70~80% | ++~+++ |
| E7 |  |  |  |  |  |
| E8-E9 | Ser Adeno Ca | 3 | 3C | 90% | ++~+++ |
| E10-E11 | Ser Adeno Ca | 3 | 4 | 80% | ++ |
| F3-F4 | Pap Ser Adeno Ca | 3 | 3C | 80% | +++ |
| F5 |  |  |  |  |  |
| F6-F7 | Adeno Ca | 3 | 3C | 80% | ++ |
| F8-F9 | Pap Ser Adeno Ca | 3 | 3C | 70% | ++ |
| F10-F11 | Pap Ser Adeno Ca | 3 | 1C | 50% | ++ |
| G3-G4 | Pap Ser Adeno Ca | 3 | 3C | 60% | ++~+++ |
| G5-G6 | Pap Ser Adeno Ca | 3 | 4 | 70% | ++~+++ |
| G7-G9 | Pap Ser Adeno Ca | 3 | 3C | 80% | ++ |
| G8 |  |  |  |  |  |
| G10-G11 | Pap Ser Adeno Ca | Borderline | 1A | 80% | ++~+++ |
| H3-H4 | Pap Ser Adeno Ca | 2 | 4 | 50% | ++ |
| H5-H7 | Pap Ser Adeno Ca | 3 | 3C | 50% | +~++ |
| H6 |  |  |  |  |  |
| H8-H9 | Pap Ser Ca | 2 | 4 | 80% | ++~+++ |
| H10-H11 | Ser Adeno Ca | 3 | 1C | 60% | + |
| I3-I4 | (HG) Pap Ser Adeno Ca | 3 | 3C | 80% | +++ |
| I5-I6 | Pap Ser Ca | 3 | 3C | 80% | ++~+++ |
| I7-I8 | Clear Cell Adeno Ca | 3 | 3C | 80% | ++~+++ |
| I9 |  |  |  |  |  |
| I10-I11 | Pap Ser Ca | 3 | 3C | 60-70% | + |

Abbreviations: Pap, papillary; Ser, serous; Adeno Ca, adenocarcinoma; Met, metastatic; Mal, malignant

| **Ovarian TMA-03-04** | | | | **NPC** | |
| --- | --- | --- | --- | --- | --- |
| **GRID** | **DIAGNOSIS** | **GRADE** | **STAGE** | **%** | **INTENSITY** |
| B3-B4 | Pap Ser Adeno Ca | 2 | 3C | --- | --- |
| B5-B6 | Pap Ser Adeno Ca | Borderline | 1A | --- | --- |
| B7 |  |  |  |  |  |
| B8-B9 | Pap Ser Cystadenocarcinoma | 2 | 3B | --- | --- |
| B10-B11 | Pap Ser Adeno Ca | 2 | 3C | --- | --- |
| C3-C4 | Adeno. Ca | 2 | 3C | 80% | ++ |
| C5 |  |  |  |  |  |
| C6-C7 | Pap Ser Adeno Ca | 3 | 3C | 60% | + |
| C8-C9 | Pap Ser Ca | 2 | 4 | 90% | +++ |
| C10-C11 | Ser Surface Pap Ca | 3 | 3C | 90% | +++ |
| D3-D4 | Pap Ser Adeno Ca | 3 | 3C | 80% | +++ |
| D5-D6 | Pap Ser Adeno Ca | 3 | 3C | --- | --- |
| D7-D8 | Pap Ser Adeno Ca | 3 | 3C | --- | --- |
| D9 |  |  |  |  |  |
| D10-D11 | Pap Ser Adeno Ca | 3 | 4 | 90% | +++ |
| E3-E4 | Pap Ser Adeno Ca | 3 | 2B | 90% | ++~+++ |
| E5-E6 | Pap Serous Adeno Ca | 3 | 3C | --- | --- |
| E7 |  |  |  |  |  |
| E8-E9 | Met Ovarian Adeno Ca | 3 | 3C | 80% | +++ |
| E10-E11 | Pap Ser Cystadenocarcinoma | 3 | 3C | 80% | +++ |
| F3-F4 | Serous Adeno Ca | 3 | 4 | --- | --- |
| F5-F6 | Pap Serous Adeno Ca | 3 | 3C | 90% | +++ |
| F7 |  |  |  |  |  |
| F8-F9 | Adeno Ca | 3 | 3C | 80% | ++~+++ |
| F10-F11 | Pap Serous Adeno Ca | 3 | 3C | 80% | +++ |
| G3-G4 | Met Ovarian Adeno Ca | 3 | 3 | 80% | +++ |
| G5 | Mucinous LMP? |  |  | 60% | ++ |
| G6-G7 | Pap Ser Adeno Ca Clear Cell | 3 | 3C | --- | --- |
| G8-G9 | Pap Ser Adeno Ca | 3 | 4 | 90% | +++ |
| G10-G11 | Endometrioid & Pap Ser Ca | N/A | 3C | --- | --- |
| H3-H4 | Mixed Mesodermal Tumor | 3 | 3C | --- | --- |
| H5-H6 | Pap Ser Ca | N/A | 3C | 90% | +++ |
| H7-H8 | Met Ovarian Adeno Ca | 3 | 3C | 80% | +++ |
| H9 |  |  |  |  |  |
| H10-H11 | Ser Surface Pap Ca | 3 | 3C | 90% | +++ |
| I3-I4 | Pap Ser Cystadeno Ca | 3 | 2A | --- | --- |
| I5-I6 | Endometrioid Ca | N/A | 3C | --- | --- |
| I7 |  |  |  |  |  |
| I8-I9 | Pap Adeno Ca | 2 | 4 | 70% | +~++ |
| I10-I11 | Pap Ser Adeno Ca | N/A | 3C | --- | --- |

| **Ovarian TMA-03-05** | | | | **NPC** | |
| --- | --- | --- | --- | --- | --- |
| GRID | **DIAGNOSIS** | **GRADE** | **STAGE** | **%** | **INTENSITY** |
| B3-B4 | Pap Ser Ca | 3 | 4 | --- | --- |
| B5 | Normal |  |  | --- | --- |
| B6-B7 | Endometrioid Ca | 2 | 1C | 50% | + |
| B8-B9 | Pap Ser Cystic Tumor | Borderline | 1C | 80% | ++~+++ |
| C3-C4 | Mucinous Adeno Ca | 1 to 2 | 3C | --- | --- |
| C5-C6 | Adeno Ca | 1 to 2 | 2C | --- | --- |
| C7 | Normal |  |  | --- | --- |
| C8-C9 | Mucinous Adeno Ca | Borderline | 3A | 90% | + |
| D3-D4 | Pap Ser Ca | 3 | 3C | 80% | ++ |
| D5 | Normal |  |  | 50% | ++ |
| D6-D7 | Pap Ser Adeno Ca | 2 | 3 | 90% | ++~+++ |
| D8-D9 | Adeno Ca | 2 | 1A | 60% | +~++ |
| E3-E4 | Pap Ser Adeno Ca | 3 | 3 | 90% | ++~+++ |
| E5-E6 | Mal Mixed Mesodermal Tumor | N/A | 3C | 60% | +~++ |
| E7 | Normal |  |  | --- | --- |
| E8-E9 | Pap Ser Ca | 3 | 1A | 90% | +++ |
| F3-F4 | Clear Cell Adeno Ca | N/A | 3C | 20% | +~++ |
| F5 | Normal |  |  | 60% | +~++ |
| F6-F7 | Pap Ser Ca | 3 | 4 | 90% | +++ |
| F8-F9 | Pap Ser Adeno Ca | 2 | 4 | 70% | ++~+++ |
| G3-G4 | Mucinous Adeno Ca | Borderline | 1A | 70% | +~++ |
| G5-G6 | Pap Ser Adeno Ca | N/A | 4 | 30% | +~++ |
| G7 | Normal |  |  | --- | --- |
| G8-G9 | Clear Cell Adeno Ca | N/A | N/A | 90% | +++ |
| H3-H4 | Pap Ser Adeno Ca | N/A | 3C | 80 | +++ |
| H5 | Normal |  |  | --- | --- |
| H6-H7 | Pap Ser Adeno Ca | 3 | 3 | 90% | ++ |
| H8-H9 | Adeno Ca/Pancreas Primary | N/A | N/A | 90% | ++ |
